# Structural and functional insights into the interaction between the bacteriophage T4 DNA processing proteins gp32 and Dda

**DOI:** 10.1093/nar/gkae910

**Published:** 2024-10-17

**Authors:** Xiaoping He, Mi-Kyung Yun, Zhenmei Li, M Brett Waddell, Amanda Nourse, Kelly A Churion, Kenneth N Kreuzer, Alicia K Byrd, Stephen W White

**Affiliations:** Department of Structural Biology, St. Jude Children’s Research Hospital, 262 Danny Thomas Place MS311, Memphis, TN 38105, USA; Department of Host-Microbe Interactions, St. Jude Children’s Research Hospital, 262 Danny Thomas Place MS221, Memphis, TN 38105, USA; Department of Structural Biology, St. Jude Children’s Research Hospital, 262 Danny Thomas Place MS311, Memphis, TN 38105, USA; Hartwell Center for Biotechnology, St. Jude Children’s Research Hospital, 262 Danny Thomas Place MS1300, Memphis, TN 38105, USA; Department of Structural Biology, St. Jude Children’s Research Hospital, 262 Danny Thomas Place MS311, Memphis, TN 38105, USA; Department of Structural Biology, St. Jude Children’s Research Hospital, 262 Danny Thomas Place MS311, Memphis, TN 38105, USA; Department of Biochemistry, Duke University Medical Center, Nanaline H. Duke Box 3711, Durham, NC 27710, USA; Department of Biochemistry and Molecular Biology, University of Arkansas for Medical Sciences, 4301 W. Markham Street Slot 516, Little Rock, AR 72205, USA; Department of Structural Biology, St. Jude Children’s Research Hospital, 262 Danny Thomas Place MS311, Memphis, TN 38105, USA

## Abstract

Bacteriophage T4 is a classic model system for studying the mechanisms of DNA processing. A key protein in T4 DNA processing is the gp32 single-stranded DNA-binding protein. gp32 has two key functions: it binds cooperatively to single-stranded DNA (ssDNA) to protect it from nucleases and remove regions of secondary structure, and it recruits proteins to initiate DNA processes including replication and repair. Dda is a T4 helicase recruited by gp32, and we purified and crystallized a gp32–Dda–ssDNA complex. The low-resolution structure revealed how the C-terminus of gp32 engages Dda. Analytical ultracentrifugation analyses were consistent with the crystal structure. An optimal Dda binding peptide from the gp32 C-terminus was identified using surface plasmon resonance. The crystal structure of the Dda–peptide complex was consistent with the corresponding interaction in the gp32–Dda–ssDNA structure. A Dda-dependent DNA unwinding assay supported the structural conclusions and confirmed that the bound gp32 sequesters the ssDNA generated by Dda. The structure of the gp32–Dda–ssDNA complex, together with the known structure of the gp32 body, reveals the entire ssDNA binding surface of gp32. gp32–Dda–ssDNA complexes in the crystal are connected by the N-terminal region of one gp32 binding to an adjacent gp32, and this provides key insights into this interaction.

## Graphical Abstract

**Graphical Abstract:**
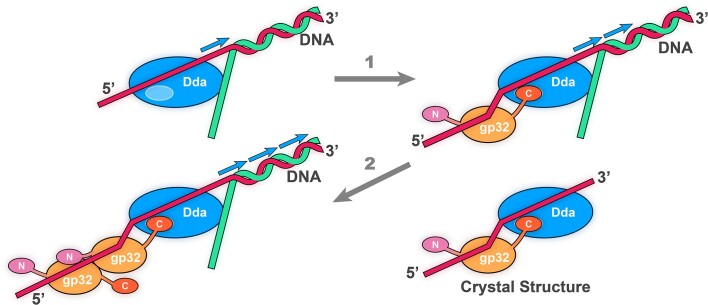

## Introduction

Phage T4 has proven to be an invaluable model system for studying the fundamental mechanisms of DNA processing. Purified T4 proteins can be combined *in vitro* to perform key DNA processes (1–3), and many of these proteins have been structurally characterized to provide insights into their mechanisms and how they interact. Structure–function studies from our group have contributed such knowledge to recombination (4–6), transcription (7–11) and the mechanism of DNA helicases (4,12,13). The single-stranded (ss) DNA-binding protein gp32 is a key player in T4 DNA processing and recruits many of the required proteins to the processing complexes. T4 proteins that are recruited by gp32 include the replicative helicase associated protein gp59 (14–20), the recombination proteins: UvsX (21) and UvsY (6), and the SF1B helicase Dda (21–23). gp32 has also been shown to have key roles in regulating the activity of the T4 RNase H nuclease (24) and in T4 replication (25).

Dda is a highly efficient monomeric helicase (26–31) that is involved in multiple T4 DNA processing scenarios (32–35). It can efficiently unwind double-stranded (ds) DNA (36–41) and also remove proteins and other adducts that associate with the dsDNA and potentially delay DNA processing (42–44). Like all SF1B helicases, Dda translocates along one DNA strand in a 5′ to 3′ direction while displacing the other strand. Our crystal structure of Dda bound to ssDNA (13) together with the previously determined structure of the SF1B helicase RecD2 (45) revealed the ratchet mechanism of the 5′ to 3′ translocation. It also showed how Dda is uniquely efficient in separating DNA strands and removing barriers (46).

gp32 comprises three domains (47): the central core region (residues 21–254) interacts with ssDNA (48,49), the N-terminal region (B domain, residues 1–20) mediates its cooperative assembly on ssDNA (50,51), and the C-terminal region (A domain, residues 255–301) recruits other T4 proteins (21,52). gp32 that lacks the N- and C-terminal regions is known as gp32-core, and gp32 that lacks the N-terminal or C-terminal regions is referred to as gp32-B and gp32-A, respectively. The crystal structure of gp32-core has been determined (53) and shown to contain an OB-fold that is present in many ssDNA- and RNA-binding proteins (54). However, there is little structural information on how the termini interact with their binding partners.

It has been demonstrated that gp32 and Dda form a complex (21–23), and studies have revealed the functional roles of this complex. Dda stimulates strand displacement during DNA synthesis in a manner that is dependent on its interaction with gp32 (22). *In vitro*, gp32 inhibits both the ATPase activity and DNA unwinding activities of Dda by competing for binding sites on the DNA substrate (32). This inhibition seems to confer substrate specificity as gp32 strongly inhibits unwinding of an inverted fork but has no effect on unwinding of a substrate mimicking a replication fork by Dda (23). During homologous recombination, Dda stimulates branch migration by UvsX with the assistance of gp32 (33), and Dda is required for stimulation of branch migration through a protein–DNA complex by UvsX and gp32 (55). Interactions of Dda with gp32 and UvsX (32,56) likely allow Dda to bind to the gp32- and UvsX-coated DNA and stimulate branch migration.

In this study, we identified a stable gp32–Dda–ssDNA ternary complex and determined its low-resolution crystal structure that clearly showed the C-terminus of gp32 bound to Dda. Guided by this structure, binding studies on peptides corresponding to fragments of the gp32 C-terminus were used to identify an optimal fragment for crystallographic analysis. This successfully yielded the structure of the C-terminal fragment bound to Dda at 3.53 Å for a detailed examination of the gp32–Dda interaction. The gp32–Dda–ssDNA complex also revealed that the ssDNA spans the two proteins, and the functional implications of this interaction were investigated by a Dda-dependent DNA unwinding assay using wild-type and mutant gp32 proteins. The structure of the gp32–Dda–ssDNA complex also provided new insights into the two surfaces on gp32-core that bind ssDNA and a partner gp32.

## Materials and methods

### Expression and purification of gp32 and Dda

The gp32 gene was cloned with an N-terminal 6xHis-tag in vector pET28b. The construct was transformed into BL21 (DE3) cells (Novagen, Madison, WI), and cells were grown in LB medium containing 20 μg/ml kanamycin at 37°C until the OD_600_ reached 0.4–0.6. The culture was grown at 16°C for 1 h before the cells were induced for 16 h by adding isopropyl β-D-1-thiogalactopyranoside (IPTG) at a final concentration of 1.0 mM before harvesting by centrifugation at 2 000 *g* for 20 min. The cell pellet was resuspended in lysis buffer (20 mM Tris-HCl pH 8.0, 100 mM NaCl, 5% glycerol, 1 mM imidazole, 5 mM β-mercaptoethanol, 0.4 mg/ml 4-[2-aminoethyl] benzenesulfonyl fluoride supplemented with EDTA-free proteinase inhibitor cocktail [Roche]) and lysed using a microfluidizer. NaCl was added to a final concentration of 400 mM, and the suspension was centrifuged at 154 000 *g* for 1 h. The supernatant was passed through a 0.45 μm syringe filter before application to a Ni^2+^ chelation column (GE Healthcare) in 20 mM Tris-HCl pH 8.0, 500 mM NaCl, 5% glycerol, 1 mM imidazole, 5 mM β-mercaptoethanol, and gp32 was eluted with a 1–200 mM imidazole gradient. Cleanup was performed by gel filtration on a HiLoad 16/60 Superdex 75 column (GE Healthcare) run in 0.5 mM EDTA, 20 mM Tris-HCl pH 8.0, 200 mM NaCl, 5 mM β-mercaptoethanol. The gp32 protein was concentrated to 5 mg/ml by Amicon® Ultra-15 Centrifugal Filters Devices (Millipore). To generate the non-His-tagged gp32 protein, the gp32 gene was cloned into the NcoI site in vector pET28a, and the transformed cells were grown, harvested and lysed as described above. Note, however, that the lysis buffer was 20 mM Tris-HCl pH 8.8, 30 mM NaCl, 5% glycerol, 5 mM β-mercaptoethanol. The protein was purified using a Q-Sepharose column (GE Healthcare) in the same buffer used for lysis and eluted with a 30–1000 mM NaCl gradient. The final cleanup by gel filtration followed the protocol used for the His-tagged protein. gp32-core (21–254), gp32-B (21–301) and gp32-A (1–254) were generated by PCR and sub-cloned with an N-terminal 6xHis-tag in vector pET28b. The constructs for the two full-length gp32 variants A255E/L262A and V258E/F265A were synthesized and cloned in vector pET28a with an N-terminal 6xHis-tag (GenScript). All gp32 variants were expressed and purified following the wild-type His-tagged gp32 protocol.

All structural and biophysical experiments involving Dda were performed using the ATPase deficient Dda mutant K38A (Dda-K38A), and expression and purification of this protein followed our previously published protocol (13). The Dda-K38A protein was concentrated to 2 mg/ml by Amicon® Ultra −15 Centrifugal Filters Devices (Millipore). Wild-type Dda was used for the DNA unwinding assays and was purified as previously described (46).

### Analysis of the gp32–Dda–ssDNA complex and its crystallization

His-tagged gp32 was used throughout unless otherwise stated. The initial identification of the complex was performed by gel filtration on a HiLoad 16/60 Superdex 75 column (GE Healthcare) run in 20 mM Tris-HCl pH 7.0, 200 mM NaCl, 0.5 mM EDTA, 5 mM β-mercaptoethanol. The same gel filtration procedure was used to prepare the gp32–Dda–ssDNA complex for crystallization, but the NaCl concentration in the running buffer was reduced to 50 mM. Equimolar amounts of gp32 and Dda–K38A were mixed with a 1.3-fold amount of oligonucleotide and a 4-fold amount of ZnCl_2_, and the purified complex was concentrated to 11 mg/ml by Amicon® Ultra-4 Centrifugal Filters Devices (Millipore). Complexes with dT16, dT17 and dT18 were prepared and crystallization trials were performed with the Protein Complex Suite (QIAGEN) screening kit using the hanging-drop procedure at 18°C. Crystals from the gp32–Dda–dT17 complex that diffracted to 6 Å were optimal and identical crystals were produced in two conditions: (i) 10% (w/v) PEG4000, 0.1 M HEPES pH 7.5, 0.1 M MgCl_2_; (ii) 25% (w/v) PEG1000, 0.1 M Na/K phosphate pH 6.5; 0.2 M NaCl. Improved crystals of the gp32–Dda–dT17 complex that diffracted to 4.98 Å were subsequently obtained using non-His-tagged gp32 and condition (ii) at 4°C.

Gel filtration using the identification protocol described above was used to prepare the gp32–Dda–dT20 complex for the AUC analysis. Equimolar amounts of gp32 and Dda–K38A were mixed with a 1.3-fold amount of dT20, and the purified complex was concentrated to 1.8 mg/ml.

### Preparation and crystallization of the gp32 C-terminal peptide/Dda/dT8 complex

Dda–K38A was added to 1.3-fold ssDNA (dT8), and the mixture was concentrated to 8 mg/ml before the further addition of 1.8-fold gp32 C-terminus peptide encompassing residues 248–270 (AATAAKKADKVADDLDAFNVDDF) prepared at a concentration of 2 mM in 10 mM HEPES pH 7.0. Crystallization trials were performed by the hanging drop procedure at 18°C using the PEGs Suite (QIAGEN). The best crystals were produced in 20% (w/v) PEG3350, 0.2 M tri-lithium citrate.

### Crystallography and data collection

The gp32–Dda–dT17 complex crystals were in space group P2_1_2_1_2_1_ and were cryoprotected in 20% ethylene glycol. The structure was determined in two steps, the first using the 6 Å resolution crystals obtained with His-tagged gp32, and the second with the 4.98 Å resolution crystals obtained with non-His-tagged gp32. In the first step, a native data set was collected at 6 Å resolution and a single-wavelength zinc SAD data set was collected at 6.8 Å. Both data sets were processed with HKL2000 (57). The structure was solved by molecular replacement (MR) with the native data using the gp32 (PDB 1GPC) and Dda (PDB 3UPU) molecules as search models. We initially found one Dda and two gp32 molecules, and combined MR-SAD phasing revealed the two expected Zn atom peaks in the gp32 molecules. This initial model, including bound oligonucleotide, was refined and optimized using the Zn phase information and reference model restraints using PHENIX (58) and COOT (59), respectively (*R*_work_/*R*_free_ = 0.36/0.43). The inclusion of the Zn SAD data considerably improved the map quality and was essential in the initial structure determination. In the second step, we collected two independent 4.98 Å native data sets that were processed with KYLIN/3DSCALE (60). Despite the data being twinned, the model was refined but the initial *R* values were high (*R*_work_/*R*_free_ = 0.38/0.47) and additional electron density suggested a missing component. Another round of MR using one of the 4.98 Å data sets and the Phaser program (61) revealed a second Dda molecule. The model was refined with the second 4.98 Å data set using twin refinement within PHENIX (58) and contains two gp32–Dda–dT17 complexes (*R*_work_/*R*_free_ = 0.2862/0.3133).

The Dda/gp32 C-terminus peptide complex crystals were in space group P3_1_21 and were cryoprotected in 25% glycerol. Data were collected at 1.0 Å wavelength to 3.53 Å and processed using KYLIN/3DSCALE (60). MR was performed by Phaser (61), which revealed one molecule in the asymmetric unit. Initial models were built and refined using COOT (59) and CNS (62). Twinning was detected in the data, and the final twin refinement was performed using PHENIX (58) (*R*_work_/*R*_free_ = 0.2528/0.2779). Note that this region of the gp32 C-terminus was included in the final refinement of the gp32–Dda–dT17 crystal structure.

Structures were validated with MolProbity (63,64) and rendered with PyMOL (version 2.5.1, Schrödinger, LLC).

### Sedimentation velocity analytical ultracentrifugation

Sedimentation velocity experiments were conducted in a ProteomeLab XL-I analytical ultracentrifuge (Beckman Coulter, Indianapolis, IN) with an AnTi-50 eight-hole rotor following standard protocols unless mentioned otherwise (65). Samples in buffer containing 20 mM Tris pH 7.0, 200 mM NaCl, 0.5 mM EDTA and 5 mM β-mercaptoethanol were loaded into cell assemblies with double sector 12 mm centerpieces and sapphire windows. The cell assemblies, containing identical sample and reference buffer volumes of 320 μl were placed in a rotor. After temperature equilibration at nominal 20°C, the rotor was accelerated to 42 000 rpm and Rayleigh interference optical data were collected. The velocity data were analyzed with the continuous sedimentation coefficient distribution model *c(s)* in SEDFIT (http://sedfitsedphat.nibib.nih.gov/software) (66). The signal-average frictional ratio and meniscus position were refined with non-linear regression and maximum entropy regularization was applied at a confidence level of *P*-0.68. The density and viscosity of the buffer at 20°C were measured using a densitometer model DMA 5000 M and a micro-viscometer model AMVn, respectively (both from Anton Paar Inc., Ashland, VA).

The velocity data of the gp32-Dda-dT20 complex were also analyzed with the 2D size-and-shape distribution model, c(*s,f/f_0_*) (with one dimension the *s-*distribution and the other the *f/f_0_*-distribution) in SEDFIT (http://sedfitsedphat.nibib.nih.gov/software), with an equidistant *f/f_0_*-grid of 0.2 steps that varies from 0.5 to 2.5 and a linear *s*-grid from 2 to 8 S with 100 s-values. The velocity data were also transformed to *c(s,f/f_0_)* and *c(s,M)* distributions with *M* the molar mass (Da), *f/f_0_* the frictional ratio, and *s* the sedimentation coefficient (*S*) and plotted as contour plots. The color temperature of the contour lines indicates the population of species (65,67).

All plots were created in GUSSI (https://www.utsouthwestern.edu/research/core-facilities/mbr/software/) (68).

### Surface plasmon resonance (SPR) experiments

#### SPR analysis 1—Dda–K38A/dT20 binding to gp32 C-terminal peptides

These experiments were conducted at 25°C using a Biacore 3000 optical biosensor (Cytiva). His-tagged Dda-K38A/dT20 was immobilized on carboxymethyldextran hydrogel-coated gold chips pre-immobilized with nitrilotriacetic acid (NTA chip; Cytiva) by capture-coupling, a hybrid method of capture and amine coupling chemistry (69). The chip was primed in chelating buffer (10 mM HEPES pH 7.4, 150 mM NaCl, 50 μM EDTA, 0.005% Tween20) and was preconditioned at 10 μl/min with three 60 s injections of wash buffer (10 mM HEPES pH 8.3, 150 mM NaCl, 350 mM EDTA, 0.05% Tween20) and one 60 s injection of chelating buffer before being charged with a 60 s injection of 500 μM NiCl_2_ in chelating buffer. After priming into immobilization buffer (20 mM HEPES pH 8.0, 150 mM NaCl, 10 mM magnesium acetate, 3 mM potassium glutamate, 1 mM TCEP, 0.05% Tween20, 10% glycerol), carboxyl groups on the hydrogel were activated with *N*-ethyl-*N*′-(3-dimethylaminopropyl) carbodiimide (EDC) and *N*-hydroxysuccinimide (NHS), and His-tagged Dda-K38A/dT20 was injected until ∼5800 RU was achieved. Any remaining active sites were blocked by Tris molecules in the binding analysis buffer (20 mM Tris-acetate pH 8.0, 150 mM NaCl, 10 mM magnesium acetate, 3 mM potassium glutamate, 1 mM TCEP, 0.05% Tween20, 10% glycerol). One flow cell on the chip was charged with Ni^2+^ and activated with EDC/NHS without adding protein to be used as a reference cell. gp32 C-terminal peptides were prepared in binding analysis buffer as a 4-fold dilution series at maximum concentration 100 μM for screening and were injected at a flow rate of 75 μl/min. A series of buffer-only (blank) injections was included throughout the experiments to account for instrumental noise. The data were processed, double-referenced and analyzed using the software package Scrubber2 (version 2.0c, BioLogic Software). The equilibrium dissociation constants (*K*_D_) were determined using steady-state analysis, specifically an equilibrium affinity analysis to fit the data to a 1:1 (Langmuir) interaction model.

#### SPR analysis 2—wild-type Dda/dT20 binding to gp32 C-terminal peptides

These experiments were conducted at 25°C using a Biacore T200 optical biosensor (Cytiva) and NTA chip sensor S series; Cytiva. The methodology was identical to that described above for surface plasmon resonance (SPR) analysis 1 apart from minor adjustments due to the change in instrumentation. These included 5 mM NiCl_2_ in the chip priming, and using a 2-fold dilution series of peptides at maximum concentration 100 μM with a minimum concentration of 0.78 μM. Also, the data were analyzed using the Biacore T200 Evaluation software package (version 3.1). The similarity in the binding of peptide 248–270, which is common to both experiments, confirms that the changes have minimal effect on the experiments.

### DNA unwinding experiments

A partial duplex substrate mimicking a replication fork was prepared by mixing equimolar concentrations of the following oligonucleotides: loading strand - TAACGTATTCAAGATACCTCGTACTCTGTACACGTTGCGATCCGACTGTCCTGCAT/36-FAM/, displaced strand - GGGATGCAGGACAGTCGGATCGCAACGTGATTTACTGTGTCATATAGTACGTGATTCAG, and leading strand - CTGAATCACGTACTATATGACACA in 10 mM HEPES/1 mM EDTA. The sample was heated to 95°C for 5 min, followed by slowly cooling to room temperature. All concentrations are final, after mixing. ATP (2.5 mM), Mg(OAc)_2_ (5 mM) and gp32 (375 nM) in assay buffer (25 mM HEPES, pH 7.5, 10 mM KOAc, 0.1 mM EDTA, 2 mM β-mercaptoethanol, 1 mg/ml bovine serum albumin and 4 mM phosphoenol pyruvate) were mixed and added to wells in one row of a black 96-well plate. The reactions were initiated by adding 25 nM substrate, 25 nM wild-type Dda and 5 U/ml pyruvate kinase/lactate dehydrogenase in assay buffer with a multichannel pipette to the entire row simultaneously. Fluorescein fluorescence was measured 8 times per minute in a VICTOR Nivo Multimode Plate Reader (Perkin Elmer) at room temperature with excitation through a 480/30 nm bandpass filter and emission measured after a 530/30 nm bandpass filter.

### Electrophoretic mobility shift experiments

Varying amounts of Dda were mixed with 5 nM 5′F-T_30_ with or without 100 nM gp32 in 25 mM HEPES, pH 7.5, 5 mM MgCl_2_, 0.1 mM EDTA, 2 mM β-mercaptoethanol, 1 mg/ml bovine serum albumin, 5% glycerol, 0.05% Orange G and either 10 mM NaCl or 150 mM NaCl. Samples were incubated at room temperature in the dark for 10 min before separating on a 6% 100:1 acrylamide:bisacrylamide gel at 4°C. Gels were imaged on an Amersham Typhoon RGB Imager (Cytiva) with excitation using a 488 nm laser and emission measured after a 525BP20 filter. Data were quantified using ImageQuant software (GE Healthcare) and fit to the Hill equation.

## Results

### Crystal structure of the gp32–Dda–dT17 complex

**Figure 1.**
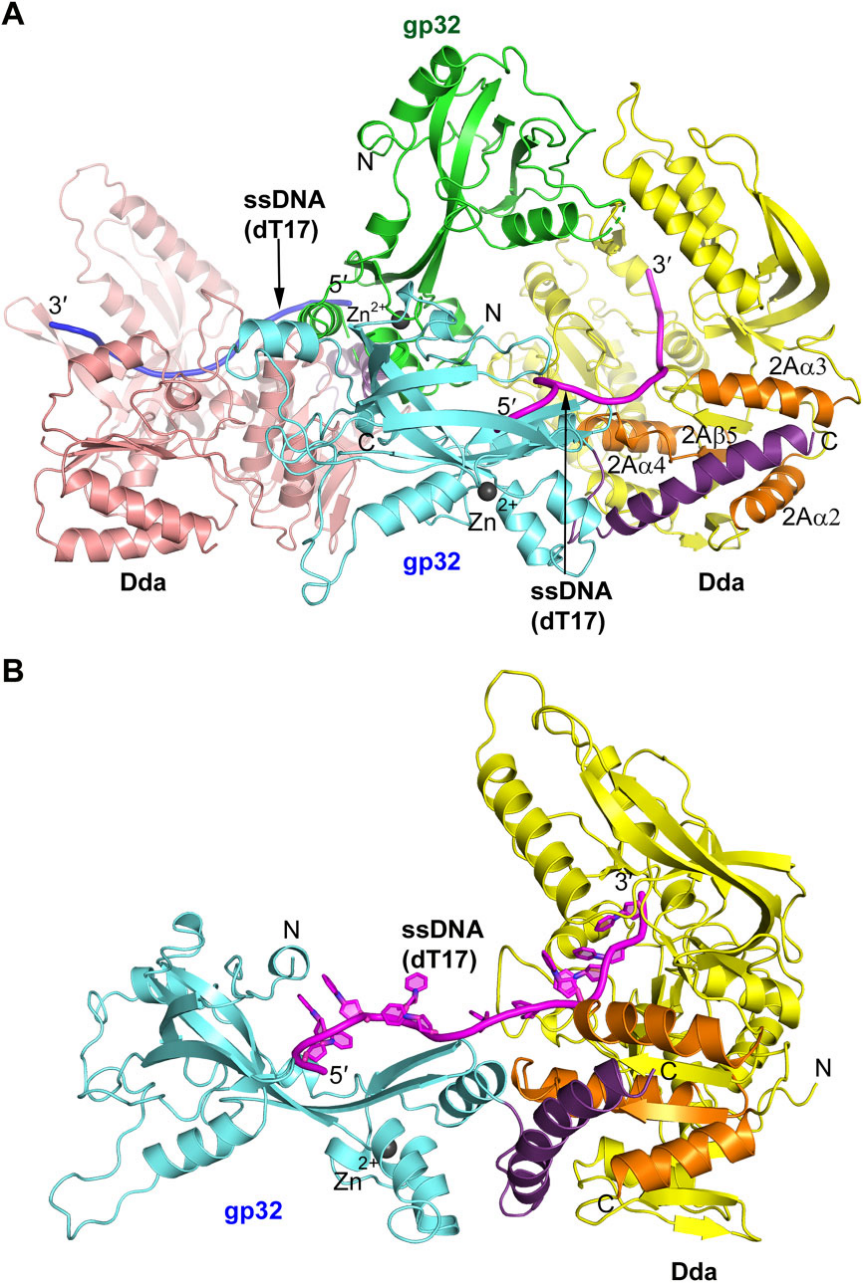
Crystal structure of the gp32–Dda–dT17 complex. (**A**) The two complexes in the crystal asymmetric unit. The gp32 molecules are shown in green and cyan, and the two Dda molecules are colored salmon and yellow. The two ssDNA molecules are shown in magenta and blue. The two Zn ions, one in each gp32, are shown as dark gray spheres. (**B**) The isolated gp32–Dda–dT17 complex. The complex shown is colored the same as the complex in (A), and the orientation is adjusted to highlight the path of the ssDNA. In each figure, the helical gp32 C-termini that engage Dda and were subsequently incorporated into the structure are shown in purple and the four secondary structures of Dda that engage the gp32 C-terminus, 2Aα2, 2Aα3, 2Aα4 and 2Aβ5, are highlighted in orange.

We have previously demonstrated (13) that the K38A active site point mutant of Dda (Dda–K38A) is more amenable to structural studies than wild-type Dda, and we continued to use Dda–K38A in the structural analyses described below. We first used gel filtration to study the interaction between full length gp32 and Dda–K38A in the absence and presence of ssDNA. When combined in the absence of ssDNA, gp32 and Dda–K38A eluted separately as confirmed by sodiumdodecyl sulfate-polyacrylamide gel electrophoresis (SDS-PAGE) analysis (Supplementary Figure S1A). gp32 eluted at a higher molecular weight than Dda, consistent with a multimeric species in solution. We then analyzed gp32 and Dda in the presence of poly dT ssDNA of increasing lengths from dT10 to dT20, and the superimposed gel filtration profiles are shown in Supplementary Figure S2. A gp32/Dda complex begins to appear in the presence dT12, and the complex is stably formed with dT16. SDS-PAGE analysis of the dT20 complex peak confirmed the presence of both proteins, and agarose gel analysis confirmed the presence of ssDNA (Supplementary Figure S1B). Encouraged by the stability of the complex, we performed crystallization trials using dT16, dT17 and dT18. Each complex was purified by gel filtration, and all yielded crystals that diffracted to low resolution. The gp32–Dda–dT17 crystals were superior, diffracting to 6 Å resolution. The structure was solved by a combination of MR using the gp32 (PDB 1GPC) and Dda (PDB 3UPU) molecules as search models, and SAD phasing using a single-wavelength zinc SAD data set collected at 6.8 Å (Table 1). We initially found one Dda and two gp32 molecules, and the SAD phasing revealed a Zn atom peak at the correct location in each of the two gp32 molecules (Supplementary Figure S3). We subsequently obtained higher quality crystals using non-His-tagged gp32 that diffracted to 4.98 Å resolution (Table 1) and identified a second Dda molecule in the structure resulting in two gp32–Dda–dT17 complexes in the asymmetric unit (Figure 1A).

**Table 1.** X-ray data collection and structure refinement statistics

Parameter	gp32–Dda–dT17	gp32–Dda–dT17	gp32 C-terminal peptide/Dda/dT8
**Data collection** ^a^			
Temperature (K)	100	100	100
Wavelength (Å)	1.000	1.27046 (Zn SAD)	1.000
Space group	P212121	P212121	P3121
Unit cell (Å) : *a*, *b*, *c*	109.86, 114,35, 147.38	109.81, 116.87, 148.46	121.79, 121.79, 88.50
Resolution (Å)	29.56–4.98 (5.08–4.98)	50.00–6.80 (7.04–6.8)	36.35–3.53 (3.59–3.53)
I/σI	12.50 (4.24)	34.92 (2.62)	4.59 (2.62)
Unique reflections	8255 (419)	3649 (348)	9276 (490)
CC1/2	1.0 (0.77)	NA	0.92 (0.29)
Rsym	0.0714 (0.3891)	0.104 (0.961)	0.1362 (0.3560)
Completeness (%)	96.83 (90.69)	99.9 (100.0)	96.22 (98.99)
Redundancy	10.56 (8.34)	9.3 (9.7)	4.84 (3.62)
**Phasing**			
Resolution (Å)	NA	6.8	NA
FOM (Figure of Merit)	NA	0.44	NA
**Refinement**			
Resolution (Å)	29.56 – 4.98	NA	36.35 – 3.53
No. of reflections	8232	NA	9237
% Complete	96.90	NA	96.25
*R*_work_/*R*_free_	0.2862 / 0.3133	NA	0.2528 / 0.2779
Rmsd from ideal values			
Bond lengths (Å)	0.004	NA	0.003
Bond angles (°)	0.790	NA	0.620
Protein residues	1326	NA	463
Nucleotides (dT)	20	NA	5
Mean B factor (Å^2^):			
Protein	200.0	NA	34.4
DNA/ion	217.1/165.6	NA	119.0
Peptide	NA	NA	21.0
Ramachandran plot:			
Favored (%)	94.28	NA	91.19
Allowed (%)	5.26	NA	8.81
Outliers (%)	0.46	NA	0.0
PDB accession code	8GME	NA	8S9I

^a^Values in parentheses refer to the highest resolution shell.

Although the resolution of the structure is low, there are four reasons why we are confident that it is correct. First, MR using the known structures of gp32 and Dda–K38A was successful. Second, there is a zinc atom in each gp32 protein at the correct location in the structure (53). Third, the structures of the two independent complexes in the asymmetric unit are very similar with only a small relative rotation of gp32 and Dda (Supplementary Figure S4). Finally, within each complex, the oligonucleotide associates with Dda–K38A at the site previously determined in the Dda–K38A crystal structure and in the same orientation (13). In one of the complexes in the asymmetric unit, there are 11 visible nucleotides in the bound oligonucleotide that span the Dda and gp32 components; five nucleotides bound to gp32, five bound to Dda–K38A and one at the interface (Figure 1B). In the second complex, there are nine visible nucleotides, but these are only bound to the Dda component. It appears that the slightly different relative orientation of gp32 and Dda compared to the first complex prevents the spanning of the oligonucleotide across the two proteins (Supplementary Figure S4).

### Sedimentation velocity analytical ultracentrifugation of Dda and gp32

**Figure 2.**
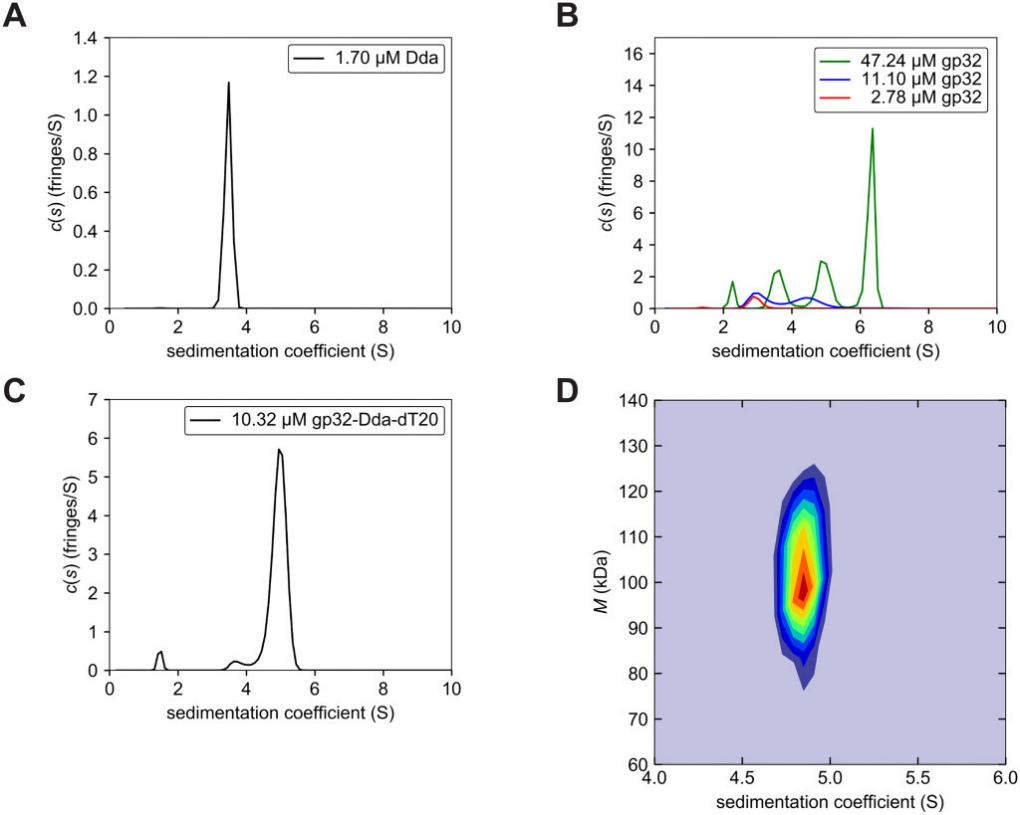
Sedimentation velocity AUC of Dda, gp32 and the gp32–Dda–dT20 complex. The sedimentation velocity profiles (fringe displacement) were fitted to a continuous sedimentation coefficient distribution model *c(s)*, as well as to a 2D size-and-shape distribution model, *c(s,f/f_0_)*. (**A**) Single *c(s)* distribution of Dda. (**B**) Superimposed *c(s)* distributions of gp32 at three concentrations. (**C**) Single *c(s)* distribution of gp32-Dda-dT20. (**D**) Velocity data of the gp32–Dda–dT20 complex analyzed with the *c(s,f/f_0_)* model and transformed to a *c(s,M)* contour plot as a heat map with increasing color temperature to maximum fringes/*S* value in red. The *s*- and mass-values are listed in Table 2.

To study the solution behaviors of Dda, gp32 and their interactions in greater detail, we used analytical ultracentrifugation (AUC). Gel filtration revealed that gp32 forms multimers in solution (Supplementary Figures S1A and S2), which is consistent with previous studies (70), and we analyzed the multimerization process in more detail. We also wanted to verify that the gp32–Dda–ssDNA complex is monomeric in solution and not dimeric as present in the crystal structure. Our previous studies on Dda have shown that Dda is monomeric in solution, and our AUC analysis clearly show that Dda-K38A at a concentration of 1.70 μM is present as a monomer in solution (Figure 2A and Table 2).

**Table 2.** Best-fit values and estimates of the *c(s)* and two-dimensional *c(s,f/f_0_*) analyses of Dda, gp32 and gp32–Dda–ssDNA complex

Sample	μM^a^	*s*_w_ (Svedberg)^b^	*s*_20,w_ (Svedberg)^c^	*M*_w_ (Da)^d^	(*f*/*f*_0w_)^e^	*R*s (nm)^f^
Dda	1.70	3.48 (90%)	3.65	60 130 (52 046)	1.45	3.78
gp32	47.24	2.27 (5%)	2.38	22 512	1.24	2.31
		3.62 (20%)	3.79	45 396	1.24	
		4.94 (25%)	5.18	72 398	1.24	
		6.31 (50%)	6.61	104 335	1.24	
gp32	11.10	3.08 (50%)	3.24	42 651	1.36	
		4.49 (50%)	4.71	74 796	1.36	
gp32	2.78	2.94 (92%)	3.09	37 323 (35 959)	1.30	2.88
gp32–Dda–dT20	10.32	4.94 (94%)	5.18	89 685	1.39	4.12
		3.76 (4%)	3.96	59 686	1.39	3.60
gp32–Dda–dT20^g^	10.32	4.84 (89%)	5.08	94 537 (94 027)^h^	1.55	4.77

^a^Total concentration of sample in micro-molar.

^b^Weight-average sedimentation coefficient *s_w_* calculated from the *c(s)* as well as the two-dimensional *c(s,f/f_0_)* distributions at 20°C with percentage protein amount in parenthesis.

^c^Standard sedimentation coefficient (*s_20,w_* -value) in water at 20°C.

^d^Weight-average molar mass values taken from the *c(s)*-transformed *c(M)* distribution as well as calculated from the two-dimensional size-and-shape model, *c(s,f/f_0_*). The theoretical molecular weights are in parenthesis.

^e^Best-fit weight-average frictional ratio values *(f/f_0_)*_w_.

^f^Stokes radius (nm)

^g^gp32–Dda–dT20 data fitted with the two-dimensional size-and-shape model, *c(s,f/f_0_*).

^h^Molar mass calculated with a weight-average partial specific volume of 0.710 ml/g.

The AUC analysis confirmed that gp32 at a concentration of 47.24 μM exists in multiple oligomeric states in solution. Upon dilution to 11.10 μM the oligomers dissociate into monomers and dimers, and at 2.78 μM all dimers dissociate into monomers (Figure 2B and Table 2). By superimposing the sedimentation coefficient distributions of gp32 at the three concentrations spanning a concentration range of 17-fold, distinct sedimentation patterns emerge with none of the peak positions at 47.24 μM overlapping with the peak positions at lower concentrations. These sedimentation patterns are characteristic of rapidly reversible concentration dependent self-associating systems where dynamically interconverting species are experimentally observed on the timescale of sedimentation (71). In contrast, a hallmark of slowly reversible systems is that the sedimentation coefficient values of the peaks stay virtually constant when comparing experiments at different concentrations spanning a large range in the vicinity of the association constant (71,72). It should be noted that, due to the dynamic nature of the interconverting species, the molar mass calculations from these analyses at high concentration do not always accurately correspond to their theoretical molar masses (72).

The Dda–K38A and gp32 solutions were then mixed with ssDNA (dT20) and the complex was purified by gel filtration. The continuous sedimentation coefficient distribution profile confirmed the formation of the complex and showed the presence of a major and a very minor peak (Figure 2C and Table 2). Further analysis of these data with the 2D size-and-shape model yielded a single peak with a molar mass of 94 537 Da (Figure 2D and Table 2). This mass is close to the theoretical molar mass of a 1:1:1 gp32–Dda–dT20 ternary complex (94 027 Da). The frictional ratio of 1.55 suggests a well-folded, moderately elongated molecular shape, consistent with the crystal structure of the complex.

### Analysis of the gp32–Dda interaction

**Figure 3.**
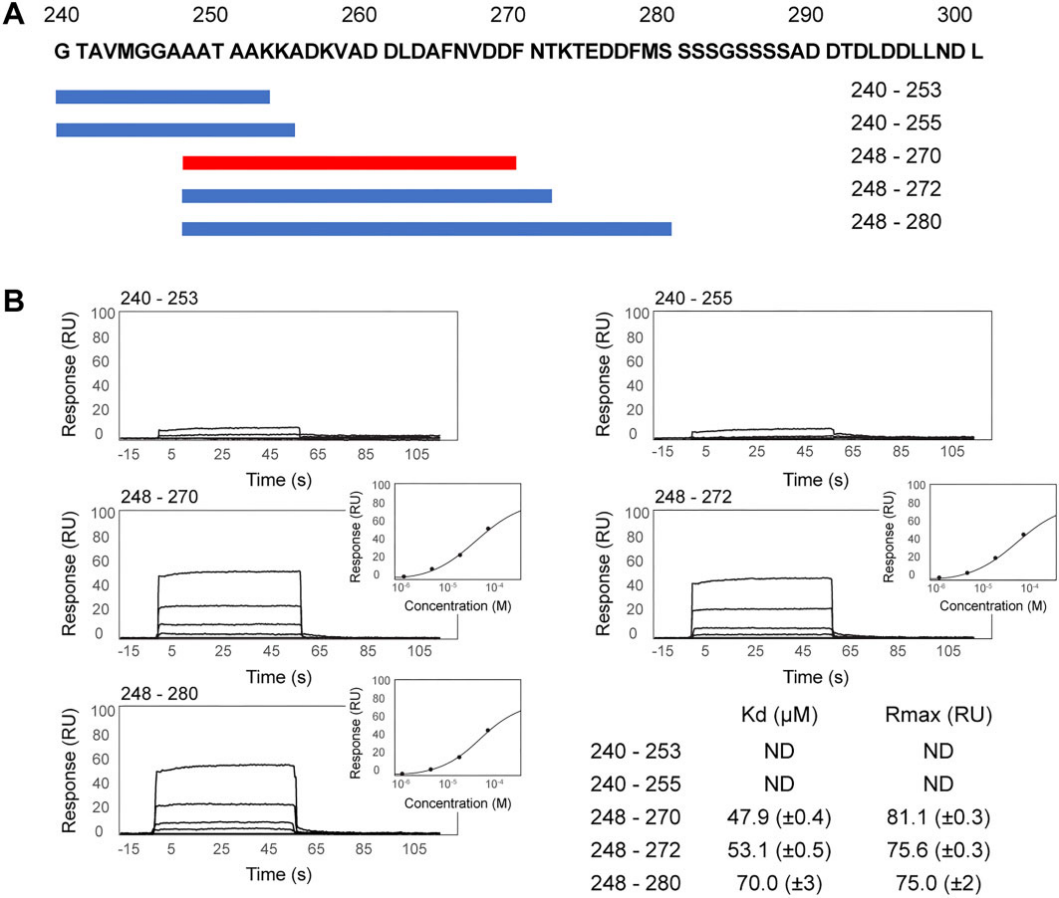
SPR analysis 1—gp32 C-terminal peptides with Dda–K38A/dT20. (**A**) The gp32 C-terminal sequence and the five (of 14 tested) peptides for which binding data are shown. (**B**) The binding data. Two peptides, 240–253 and 240–255, showed no binding. Three peptides, 248–270, 248–272 and 248–280, show similar robust binding, and 248–270 is slightly superior. The response curves (insets) for 248–270, 248–272 and 248–280 were used to calculate their indicated binding affinities.

Within each complex in the crystal structure and at the same location, there is a region of unassigned electron density that is ideally positioned to be the C-terminus of gp32 interacting with its partner Dda–K38A. The two electron densities are very similar and consistent with an α-helix that engages a surface cavity on the 2A RecA subdomain of Dda comprising helices 2Aα2, 2Aα3 and the loop connecting strand 2Aβ5 and helix 2Aα4. To analyze this interaction in more detail, we used SPR to first establish whether C-terminal peptides of gp32 can bind to Dda and then to identify the minimal C-terminal peptide that is required to form a tight complex for structural analysis. In this SPR experiment (SPR analysis 1), Dda-K38A/dT20 was captured on the chip and 14 synthetic peptides spanning residues 240–280 of the gp32 C-terminus were tested for binding. Residues 281–301 at the very C-terminus contain multiple serine and aspartic acid residues that we considered unlikely to be involved in specific interactions with Dda and were not included in the initial analysis. The key results from five of the peptides are shown in Figure 3. Peptides 240–253 and 240–255 failed to bind, but peptides 248–270, 248–272 and 248–280 bound robustly. Peptide 248–270 was the optimal shortest peptide and bound with a *K*_D_ of 47.9 (±0.4) μM.

**Figure 4.**
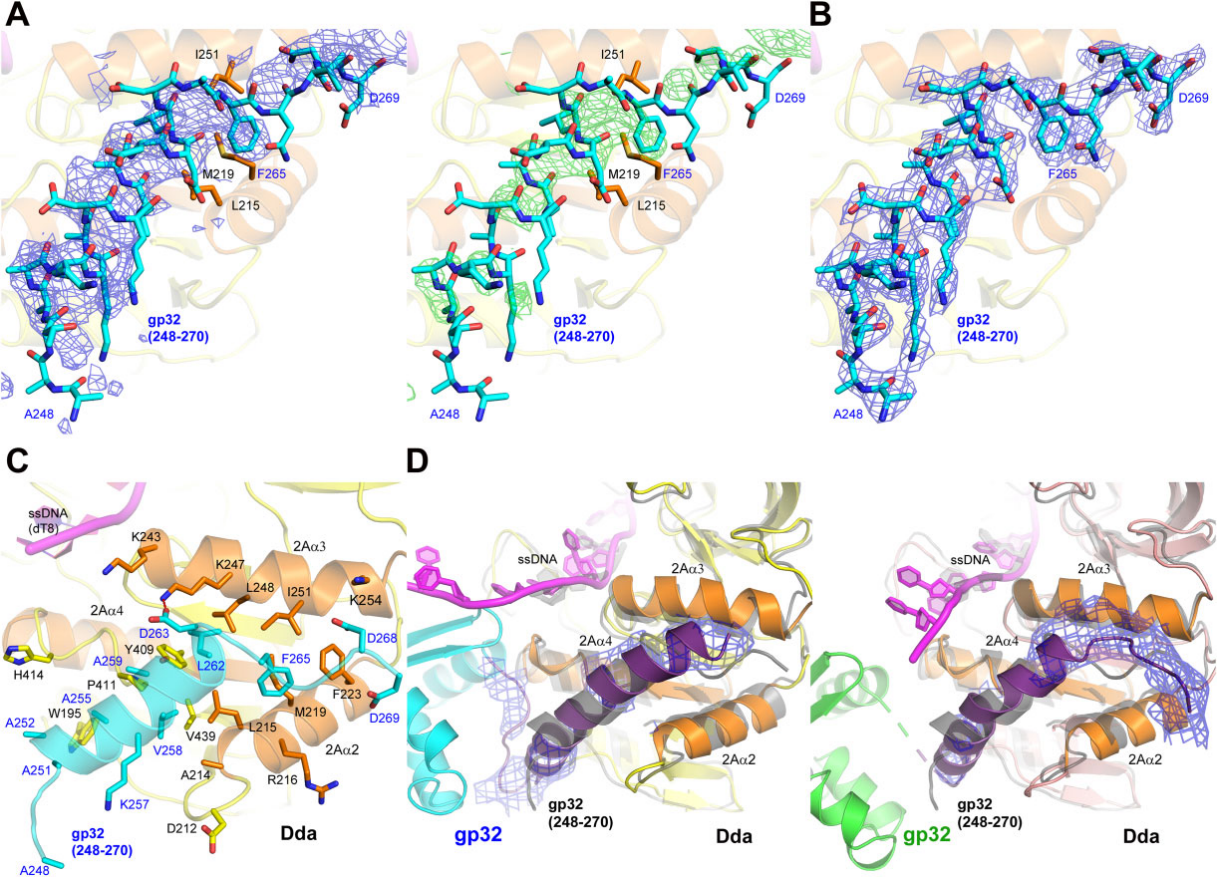
The C-terminal region of gp32 bound to Dda. (**A**) The initial unbiased electron density maps of the bound peptide in the gp32 C-terminal peptide/Dda/dT8 complex. The 2Fo-Fc electron density is shown as a blue mesh contoured at 1σ (left) and the Fo-Fc electron density is shown as a green mesh contoured at 2σ (right). (**B**) The final refined 2Fo-Fc electron density map of the bound peptide, which is superimposed (cyan carbon sticks). The electron density is shown as a blue mesh contoured at 1σ. The final structure of the peptide is also shown in (A) to demonstrate the quality of the initial maps. (**C**) The C-terminal region of gp32 bound to Dda as revealed in the final refined crystal structure of the gp32 C-terminal peptide/Dda/dT8 complex. The bound peptide and the carbon atoms of the interacting residues are cyan, and the interacting Dda secondary structures 2Aα2, 2Aα3, 2Aα4 and 2Aβ5 and the carbon atoms of the interacting residues are orange. (**D**) The C-termini in the final refined crystal structure of the gp32–Dda–dT17 complex. There are two independent interactions with Dda in the dimeric complex, and both are very similar. The orientations are identical and match that shown in Figure 1B. The 2Fo-Fc electron densities of the gp32 C-termini are unbiased and were calculated without the fitted C-termini in the gp32-Dda-dT17 complex; they are shown as blue mesh contoured at 1σ. The superimposed structure of the gp32 C-terminal peptide/Dda/dT8 complex (gray color) is shown in gray. In (C) and (D), the coloring of the proteins and ssDNA match that shown in Figure 1, and the interacting Dda secondary structures 2Aα2, 2Aα3, 2Aα4 and 2Aβ5 are highlighted in orange.

Peptide 248–270 was selected for crystallization trials with Dda–K38A and dT8. Crystals were obtained that diffracted to 3.53 Å and the initial refinement clearly showed the expected Dda/dT8 complex and additional electron density consistent with the bound peptide at the same location of Dda seen in the gp32–Dda–dT17 crystal structure (Figure 4A). The peptide was fitted into the electron density and the peptide-Dda/dT8 was successfully refined with *R*_work_/*R*_free_ values of 0.2528/0.2779 (Figure 4B and Table 1). Details of the Dda–peptide interaction are shown in Figure 4C. Residues 250–264 of the peptide are α-helical, and the termini have no secondary structure. Val258, Leu262 and Phe265 appear to be key in stabilizing the complex: Val258 interacts with Dda residues Ala214, Leu215, Tyr409, Pro411 and Val439; Leu262 interacts with Leu215, Lys247, Leu248, Ile251 and Tyr409; Phe265 interacts with Leu215, Arg216, Met219 and Ile251. In addition, Asp263 on the peptide forms a salt bridge with Lys247 and possibly Lys243, and alanines 251, 252, 255 and 259 on the peptide create the necessary space for Dda residues Trp195, Pro411 and His414.

To confirm that the peptide–Dda–dT8 complex structure is consistent with the corresponding structure within the low resolution gp32–Dda–dT17 crystal structure, we compared the complex with the unbiased 2Fo-Fc electron densities of the gp32 C-termini in the two copies of the peptide–Dda–dT8 complex in which the C-termini had not been fitted. The agreement was excellent (Figure 4D). Based on this analyses, we incorporated the higher resolution structure of the peptide–Dda interaction into the final refinement of the low resolution gp32–Dda–dT17 structure, specifically residues 240–269 in one complex and residues 246–270 in the second complex (Table 1 and Figure 1; Supplementary Figure S4). The inclusion of these residues resulted in a small but significant improvement in the *R*_work_/*R*_free_ values (0.2964/0.3218 to 0.2862/0.3133). The electron density of one of the complexes in the final crystal structure is shown in Supplementary Figure S5. In this final structure of the gp32–Dda–dT17 complex, there was no obvious additional electron density corresponding to residues 271–301 at the very C-terminus of gp32, and they appear to be disordered.

Because the gp32–Dda complex appears to be stabilized predominately by hydrophobic interactions, we tested the effect of gp32 on Dda affinity for ssDNA at high salt. Dda unwinding activity is inhibited at high salt with 75–200 mM NaCl resulting in significant inhibition of activity (23,32). Dda affinity for ssDNA is also greater at low salt than at high salt (Supplementary Figure S6A, S6C and S6D). As predicted, addition of gp32 increases the affinity of Dda for the ssDNA. Interestingly, gp32 also reduces the fraction of ssDNA bound by Dda (Supplementary Figure S6B, S6Cand S6D), likely because gp32 and Dda compete for binding to the same sites on ssDNA.

### DNA unwinding by Dda and gp32 variants

**Figure 5.**
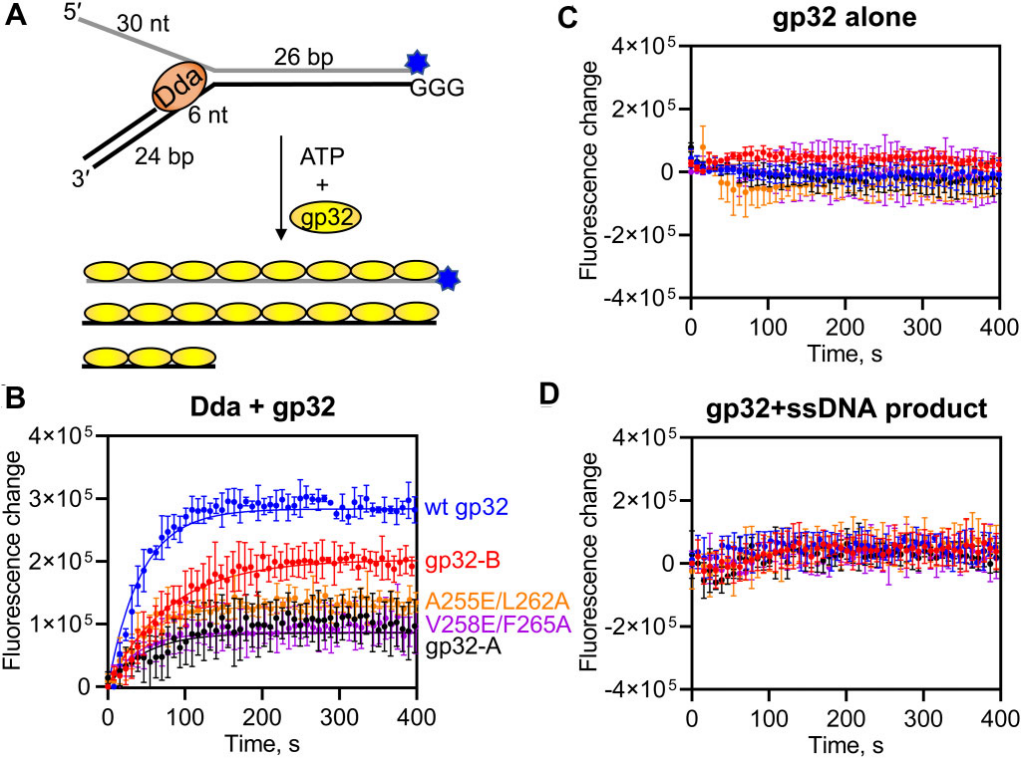
Interactions of the N- and C-termini of gp32 are necessary for gp32 to effectively trap ssDNA produced by Dda. (**A**) The fluorescence of the partial duplex substrate is quenched by the three guanine residues on the displaced strand. In the presence of gp32 and ATP, Dda unwinds the substrate producing ssDNA which is trapped by gp32. (**B**) The fluorescence intensity is indicative of product formation. Data were fit to a single exponential function. Rate constants are 0.023, 0.013, 0.021, 0.023 and 0.026 s^−1^ for reactions containing wild-type gp32, gp32-B, gp32-A, gp32 A255E/L262A and gp32 V258E/F265A, respectively. Amplitudes of the product formation curves are 280 000, 200 000, 86 000, 130 000 and 87 000 for reactions containing wild-type gp32, gp32-B, gp32-A, gp32 A255E/L262A and gp32 V258E/F265A, respectively. Standard deviations of triplicate experiments are shown. (**C**) Fluorescence change from unwinding reactions lacking Dda. (**D**) Fluorescence change from mixing gp32 with the ssDNA product strands.

The arrangement of gp32, Dda and ssDNA in the complex crystal structure (Figure 1B) suggests that, as Dda unwinds duplex DNA, the ssDNA would be ‘fed’ to the ssDNA binding site of gp32 where it would be sequestered in a ssDNA conformation. This suggested that the annealing trap that is normally included in helicase reactions to prevent reannealing of the ssDNA products could be omitted if gp32 was included with Dda to prevent reannealing of the ssDNA products. Using a quenched substrate with three guanine residues opposite a fluorescein label (73) (Figure 5A), we observed an increase in fluorescence due to unwinding of the duplex upon addition of ATP and gp32 (Figure 5B). The amplitude of product formation is an indicator of the equilibrium between dsDNA substrate and ssDNA products, with a higher amplitude indicating that the gp32 variant is more effective at preventing reannealing of the ssDNA product strands. Wild-type gp32 is more effective at trapping the products than either gp32-B or gp32-A (Figure 5B). Two gp32 variants (A255E/L262A and V258E/F265A) that were designed based on the Dda–peptide structure to reduce gp32–Dda interactions behaved similarly to gp32-A (Figure 5B). Addition of gp32 alone to either the substrate or ssDNA product does not result in a similar fluorescence increase (Figure 5C,D). Thus, gp32 containing both the N- and C-terminal helices effectively sequesters ssDNA as it emerges from Dda, as predicted by the structure of the complex.

**Figure 6.**
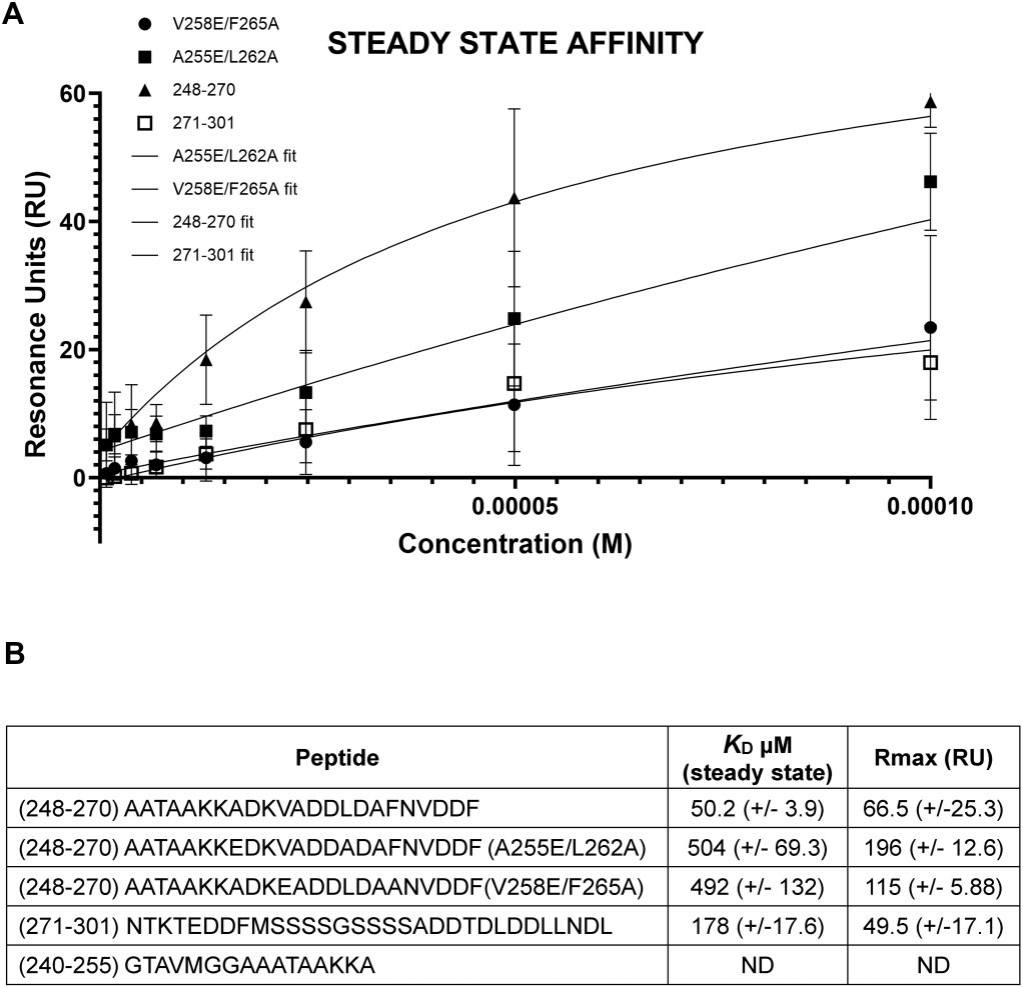
SPR analysis 2—gp32 C-terminal peptides with wild-type Dda/dT20. (**A**) Steady-state binding profiles of peptide 248–270 (positive control), the mutant peptides A255E/L262A and V258E/F265A derived from peptide 248–270, and peptide 271–301, which encompasses most of the acidic residues in the gp32 C-terminus. The steady-state data presented includes the average of at least three independent experiments for each peptide, and the fitting is based on these averages. The graphical SPR sensorgram steady-state data reflect this average. (**B**) Table showing the peptide sequences, the steady-state binding affinities (*K*_D_ in μM), and the *R*_max_ values. This table represents the average of three experiments, from which the *K*_D_ values were also determined. Peptide 240–255 serves as a negative control based on SPR analysis 1 (Figure 3) and shows no binding in this analysis. For additional details, see Supplementary Figure S7.

To support these functional studies, we performed a second set of SPR experiments using wild-type Dda bound to dT20 (SPR analysis 2). Guided by the SPR data shown in Figure 3, we tested four gp32-derived peptides; 248–270 as a positive control, 240–255 as a negative control, and the A255E/L262A and V258E/F265A mutants of 248–270. The results are shown in Figure 6 and Supplementary S7. As previously observed, the negative control fails to bind and the positive control binds robustly, which also confirms that the K38A Dda active site mutation has no effect on peptide binding. However, the two mutant peptides bind ∼10-fold less than the wild-type peptide, which is consistent with the functional results (Figure 5B). As with the previous SPR analysis, we specifically analyzed the steady-state binding characteristics and observed that steady-state saturation was not achieved with the mutant peptides. This confirms that the affinity of the mutant peptides for Dda is significantly weaker compared to the wild-type 248–270 peptide. Specifically, the lack of steady-state saturation indicates that the interaction between the mutant peptides and Dda is less stable and that a lower proportion of the peptide–Dda complex is formed at equilibrium. We also took the opportunity to evaluate the binding of peptide 271–301, which includes most of the acidic residues within the gp32 C-terminus (Figure 3A). Although binding was detected (Figure 6 and Supplementary Figure S7), the SPR profile for this peptide also indicates a significantly reduced affinity compared to the 248–270 peptide.

### The binding site on gp32-core for ssDNA

**Figure 7.**
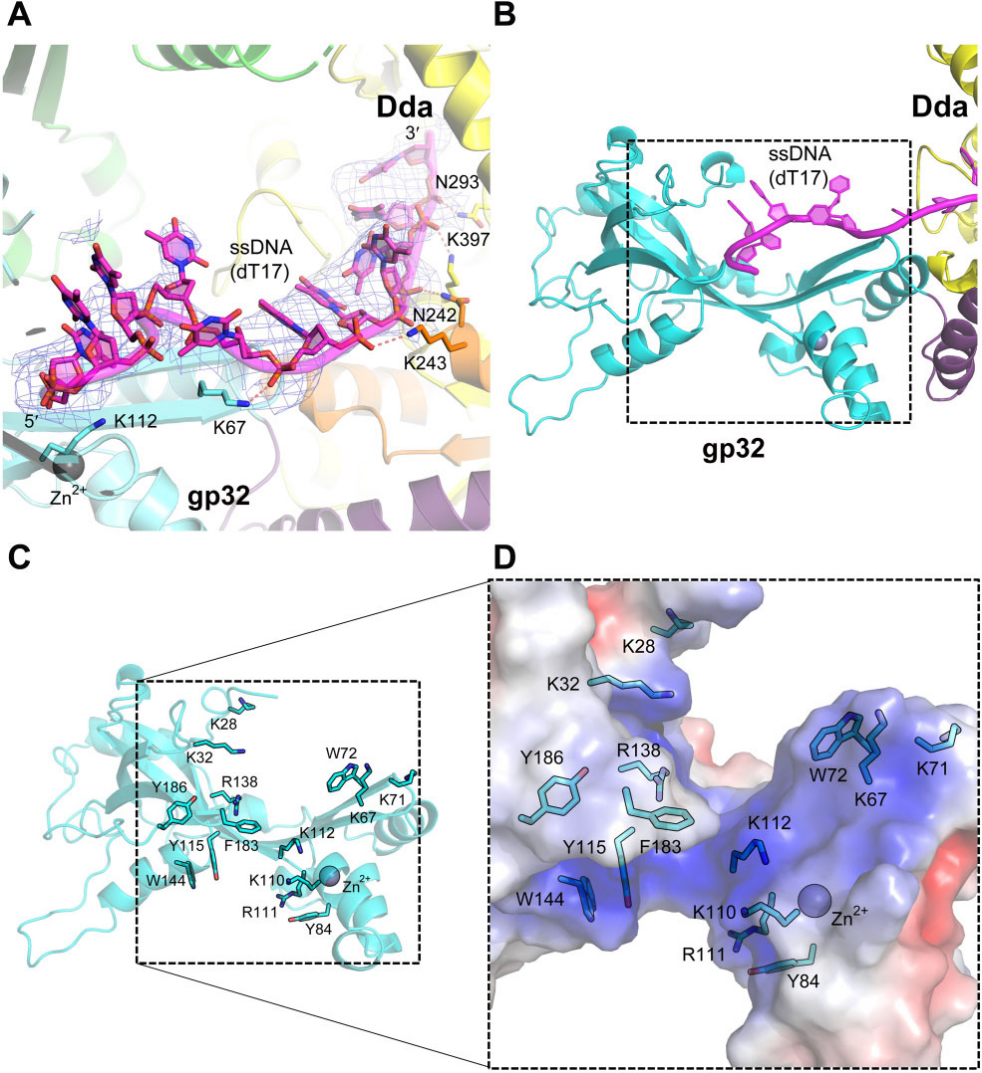
The ssDNA-binding site of the gp32 core region. (**A**) The ssDNA 2Fo-Fc electron density (light blue mesh) for the gp32–Dda–dT17 complex shown in Figure 1B contoured at 1σ. The coloring matches that shown in Figure 1B and some key residues are indicated. (**B**) Boxed region of gp32 that interacts with ssDNA as revealed in the gp32–Dda–dT17 complex and the previously determined crystal structure of the gp32 core (PDB 1GPC) (51). (**C**) The exposed basic and aromatic residues of the boxed region of gp32 shown in (B) that create the ssDNA binding surface. The residues are from two structures: the gp32–Dda–dT17 complex shown in Figure 1B and those previously identified from the crystal structure of the gp32 body (51). (**D**) The electrostatic potential surface within the boxed region of gp32 shown in (B). The potential (from −5 to 5 kT/e) was rendered using the Adaptive Poisson-Boltzmann Solver (APBS) package within PyMOL; red is negative potential, blue is positive, and white is neutral/hydrophobic. Panels (C) and (D) were generated from the crystal structure of the gp32 body (PDB 1GPC) (51).

As noted above, in the gp32–Dda–dT17 crystal structure, one of the complexes in the asymmetric unit reveals that the bound oligonucleotide spans and engages both proteins (Figures 1B and 7A). Despite the low resolution, the oligonucleotide binding site on the gp32 core domain is unequivocal (Figure 7B) and comprises Lys28, Lys32, Lys67, Lys71, Trp72, Lys112, Arg138 and Phe183 that engage the five bound nucleotides. The weakly bound ssDNA in the crystal structure of the gp32 core domain (53) also revealed a positive surface patch containing exposed aromatic residues ideal for accommodating ssDNA comprising Trp72, Tyr84, Lys110, Arg111, Tyr115, Trp144, Phe183 and Tyr186. The two patches are adjacent, with Trp72 and Phe183 common to both, and we suggest that the two patches combined reveal the entire path of the ssDNA through the gp32 core domain. The combined binding surface is shown in Figure 7C, and its positively charged surface potential is shown in Figure 7D.

### The binding site on gp32-core for the gp32 N-terminal region

In the crystal structure of the gp32–Dda-dT17 complex, interactions between adjacent gp32 molecules have important roles in creating and stabilizing the crystal lattice. One of these interactions is within the asymmetric unit dimer and the second interaction is between symmetry-related gp32 proteins (Supplementary Figure S8A). Unassigned electron density associated with each of the two independent gp32 molecules in the structure is consistent with the gp32 N-terminus (Supplementary Figures S5 and S8A). In both cases, the electron density is consistent with an α-helix positioned adjacent to the body of the partner gp32 and interacting with the α-helix spanning residues 141–152 (Supplementary Figure S8B). However, the gp32 monomers within two dimers are in different relative orientations (Supplementary Figure S8B), which presumably reflects their flexible association, at least in the absence of bound ssDNA. This interaction appears to be very stable because it occurs in solution despite the presence of the N-terminal His-tag on gp32. Thus, the assay shown in Figure 5 requires the gp32–gp32 interaction, and the gp32 oligomers identified by gel filtration and AUC presumably also require the interaction.

## Discussion

gp32 was first characterized over 50 years ago (74) and has served as a paradigm for the functionally homologous proteins that exist in eukaryotes and prokaryotes. Despite much work on gp32, including the crystal structure of the core domain (53), the ways in which the N- and C-termini recruit their partners were not known. This work focused on the structural basis of the C-terminus interactions and specifically on its interaction with T4 helicase Dda that is a known binding partner. We identified and purified a gp32–Dda–ssDNA complex, obtained crystals and determined the structure to 4.98 Å resolution. AUC analyses confirmed that the complex is present in solution. Despite the low resolution, the structure and subsequent SPR analyses allowed us to identify an optimal C-terminal gp32 peptide that binds tightly to Dda, and the complex was structurally characterized by crystallography. The structure of the gp32–Dda–ssDNA complex also provided new insights into the two distinct surfaces of the gp32 core that bind ssDNA and the N-terminal region of a partner gp32.

The failure to generate higher resolution crystals of the gp32–Dda–ssDNA complex that we identified in solution was disappointing, but the final structure offers several explanations. First, apart from the gp32 C-terminal interaction and the spanning ssDNA interaction observed in one of the complexes in the asymmetric unit, the gp32 and Dda proteins have limited contact (Figure 1B). Second, there are two gp32 dimers in the crystal lattice within and between asymmetric units, both of which appear to be linked by the N-terminus, and these create a filament in the crystal with Dda molecules arrayed on the outside (Supplementary Figure S8A). This filament has the potential to significantly increase the flexibility of the crystal lattice. Third, the interactions of the gp32 N- and C-termini with their binding partners appear to be quite flexible. Thus, the relative orientations of gp32 and Dda within the two complexes of the asymmetric unit are very similar but still significantly different (Supplementary Figure S4), and the relative orientations of the gp32 monomers in the two independent gp32 dimers are quite different (Supplementary Figure S8B). Finally, the Dda–ssDNA complex appears to be inherently quite flexible. The original structure was only determined at 3.3 Å (13), and the structure presented here bound to the gp32 C-terminal peptide was determined at 3.53 Å. There were three molecules in the asymmetric unit of the original structure, and we noted some variability consistent with a degree of flexibility (13). Comparison of these three molecules with the peptide-bound structure also shows variability, particularly in the ‘pin’ region (1B, residues 86–107) that has a quite different conformation. The RMSD values on 436 Cα atoms including the pin are 3.2–3.5 Å (3.498, 3.347 and 3.248 Å) and on 414 Cα atoms without the pin are 1.5–1.8 Å (1.792, 1.565 and 1.534 Å). Note that the pin conformation in the peptide-bound structure partially occludes the ssDNA-binding groove, which explains why only three nucleotides are bound.

Despite the challenging low resolution structure determination of the gp32–Dda–ssDNA complex, there are a number of compelling reasons why we are confident that it is correct. The structures of the gp32 core domain and Dda components completely agree with their individual crystal structures, the two independent complexes in the asymmetric unit are almost identical, the two gp32 C-termini engage their Dda partners in the same way and very similar to that revealed in the structure of the isolated complex, and the final R factors are consistent with a structure at this resolution (Table 1). Also, the ssDNA-binding surface of gp32 revealed by the gp32-Dda-ssDNA complex is consistent with that derived from the crystal structure of the gp32 core domain, and indeed complements it by extending the binding surface that is heavily populated by basic and aromatic residues. However, the biological relevance of the two complexes in the crystal asymmetric unit is questionable, and the dimer appears to be a crystallization artifact resulting from the gp32–gp32 interaction mediated by the N-terminal region.

We also note that the gp32–Dda–ssDNA complex is completely consistent with its biological role. In one of the complexes in the crystallographic dimer, the bound oligonucleotide spans the Dda and gp32 components and is consistent with Dda directly ‘feeding’ the ssDNA to the ssDNA-binding site of gp32 (Figure 1B). Dda is an extremely efficient helicase, and this organization would allow the generated ssDNA to be quickly sequestered by gp32. Our duplex DNA unwinding data are fully consistent with this idea. We observed that wild-type gp32 effectively sequestered the ssDNA products produced by Dda. gp32-B is less effective at trapping the ssDNA. Considering that gp32-B has reduced affinity for ssDNA (47) and is defective in cooperative binding to ssDNA (75), this is not surprising. A previous study from the Morrical laboratory (23) found that gp32-B stimulated unwinding by Dda on a similar substrate, suggesting that formation of stable gp32 filaments may prevent Dda from accessing the ssDNA. These studies contained a peptide nucleic acid (PNA) trapping strand to prevent reannealing of the products, so the measured effect of gp32 is due entirely on the interaction with Dda and is independent of the ability of gp32 to trap the ssDNA products produced by Dda. The results in Figure 5 are dependent on both the interaction of Dda with gp32 and the ability of gp32 to protect the ssDNA from access by a complementary ssDNA strand, likely contributing to the different results with the gp32-B variant. gp32-A lacks the C-terminus that interacts with Dda but retains full ssDNA binding activity (48), and is even less efficient at trapping the ssDNA products. Wild-type gp32 and gp32-B may be recruited to the ssDNA as it emerges from Dda via the C-terminal tail. The lack of the C-terminal tail in gp32-A would prevent its recruitment by Dda and delay its binding to the ssDNA, resulting in reannealing of the single-strands behind Dda. In addition, Dda is not very processive (39) and likely requires multiple binding events to unwind the duplex. Dda may therefore be able to invade a gp32 filament with the C-terminal tails better than a filament of gp32-A, with which it cannot interact. A reduction in Dda binding to gp32-A coated ssDNA would reduce the ability of Dda to unwind the substrate. We note that these possibilities are not mutually exclusive, and both require gp32–Dda interactions visualized in our structures for gp32 to effectively sequester the ssDNA produced by Dda as observed in our unwinding data.

Finally, a number of studies have suggested that the C-terminal region of gp32 binds the ssDNA binding site in the absence of ssDNA (70,76,77). The C-terminal region is typically labeled ‘acidic’, but the acidic residues are mainly located at the very C-terminus (273–301) that are not visible in the gp32–Dda–ssDNA complex and therefore unlikely to be involved in the interaction with Dda. This suggests that the C-terminal region may be bifunctional and divided into two separate regions; ∼248–269 that interacts with recruited proteins such as Dda, and ∼273–301 that is involved in the ssDNA-binding functions of gp32. Their adjacency further suggests that these functions are mutually exclusive. We did observe by SPR (Figure 6) that the acidic region interacts weakly with Dda when presented as an isolated peptide. In the SPR analysis, ssDNA (dT20) is present and should be occupying the ssDNA-binding groove of Dda. The peptide is unlikely to displace this ssDNA, but it may instead engage the positively charged surface patches that we previously identified on Dda and suggested interact with other regions of the DNA substrate (13).

## Supplementary Material

gkae910_Supplemental_File

## Data Availability

Crystal structures have been deposited in the PDB database (https://www.rcsb.org/) under entry IDs 8GME, 8S9I.
