# Supplementary material for: Structural and functional insights into the interaction between the bacteriophage T4 DNA processing proteins gp32 and Dda
Source: Nucleic Acids Res. 2024 Oct 17;52(20):12748–62. doi: 10.1093/nar/gkae910 (PMC11551737; doi:10.1093/nar/gkae910)
Supplement: gkae910_Supplemental_File [file gkae910_supplemental_file.pdf]

Supplementary Materials for:

Structural and Functional Insights into the Interaction between Bacteriophage T4 DNA Processing Proteins gp32 and Dda

Xiaoping He, Mi-Kyung Yun, Zhenmei Li, M. Brett Waddell, Amanda Nourse, Kelly A. Churion, Kenneth N. Kreuzer, Alicia K. Byrd and Stephen W. White

Correspondence to: stevew.white@stjude.org

This PDF file includes:

Figures S1 to S8

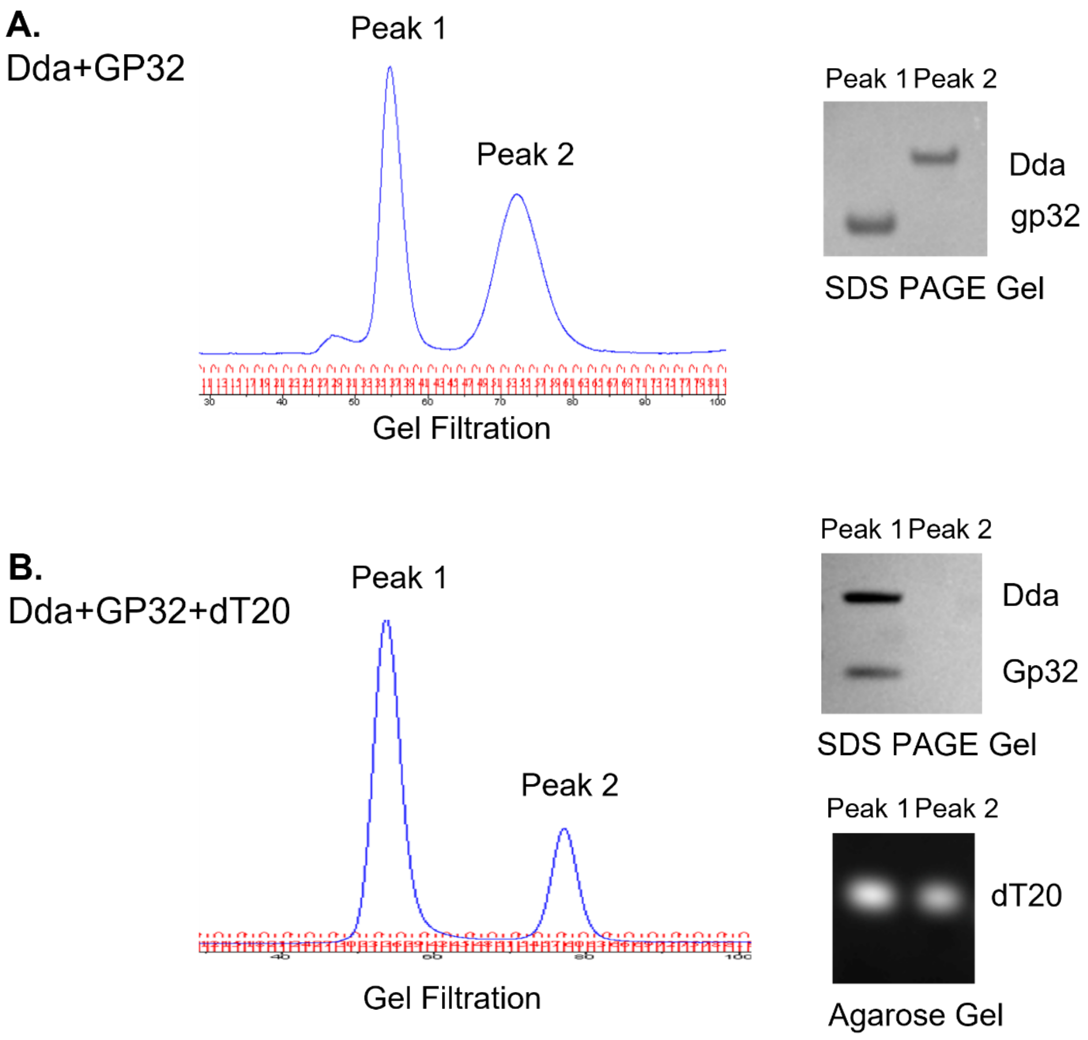

Figure S1. Comparison of gp32/Dda mixtures in the absence and presence of dT20 by gel filtration, SDS-PAGE and agarose gel. **(A)** In the absence of dT20, gp32 (peak 1) and Dda (peak 2) elute separately, and gp32 elutes at a position consistent with a multimer. **(B)** In the presence of dT20, all three components elute as a single peak (peak 1), with excess dT20 appearing in a second peak (peak 2).

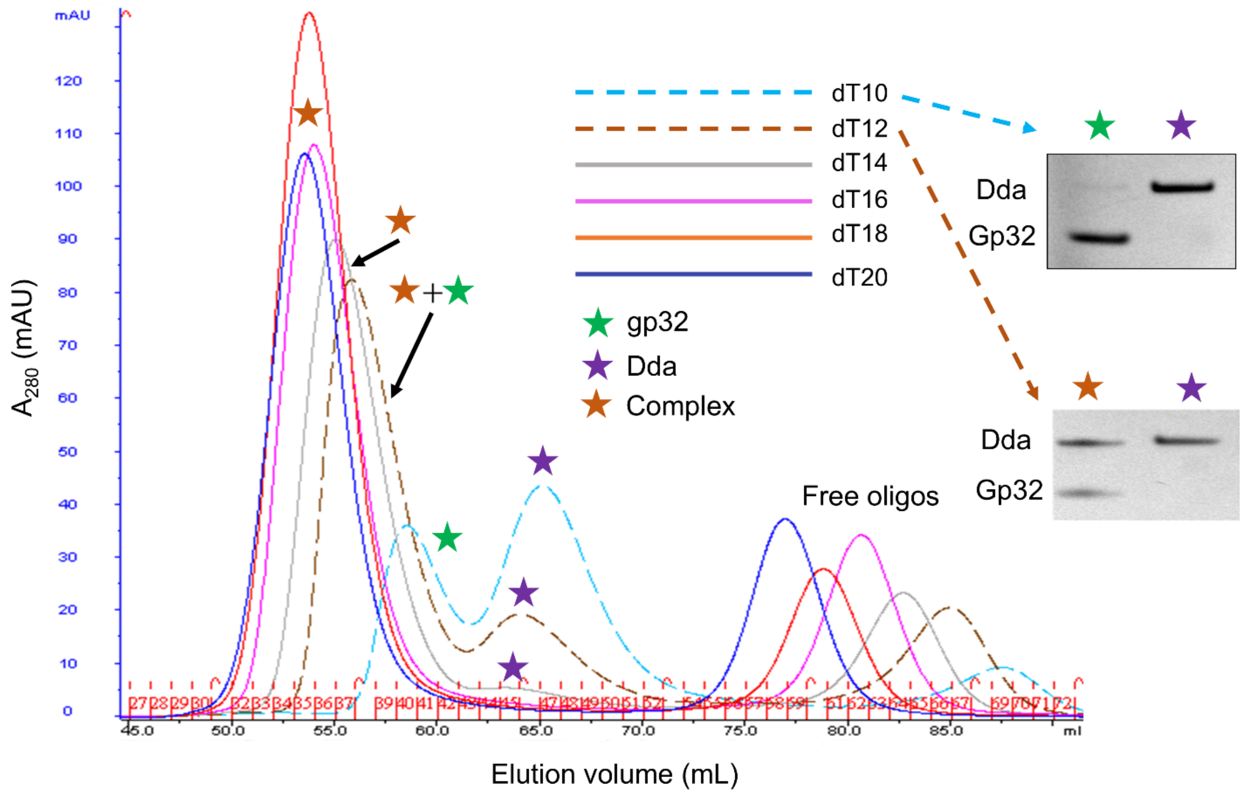

Figure S2. Analysis of gp32, Dda and dT oligonucleotide mixtures by gel filtration. Superimposed are gel filtration profiles using oligonucleotides of increasing length from dT10 to dT20. For dT10 (dotted cyan), there are separate peaks for gp32 and Dda as confirmed by SDS-PAGE. For dT12 (dotted brown), the Dda peak is reduced, and a higher molecular weight ‘complex’ peak appears that contains both gp32 and Dda, as confirmed by SDS-PAGE. Note that the shape and position of the dT12 complex peak combined with the presence of free Dda suggests that the complex peak is actually a combination of complex and free gp32. For dT14 (solid grey), the complex peak is more symmetrical and there is very little free Dda, which suggests that the majority of gp32 and Dda are present in a stable complex. For dT16 (solid magenta), dT18 (solid orange) and dT20 (solid blue) there are well-defined complex peaks with a consistent molecular weight (elution position) and no free Dda. The presence of oligonucleotides in the complexes and in the free oligonucleotide peaks was confirmed by agarose gels (not shown but see Figure S1B for dT20).

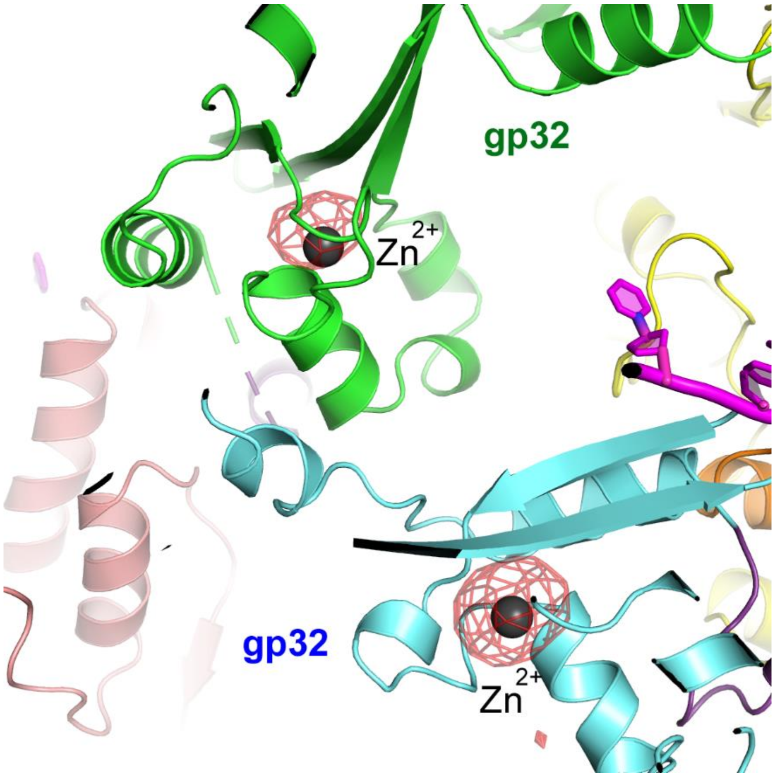

Figure S3. Identification of Zn ions. Anomalous map calculated from data collected at 1.27046 Å on the Dda-gp32-dT17 complex crystal at 6.8 Å resolution. The anomalous map is rendered in red and contoured at 3.0σ, and clearly shows the position of a Zn atom at the same location in each gp32 molecule in the two complexes in the asymmetric unit.

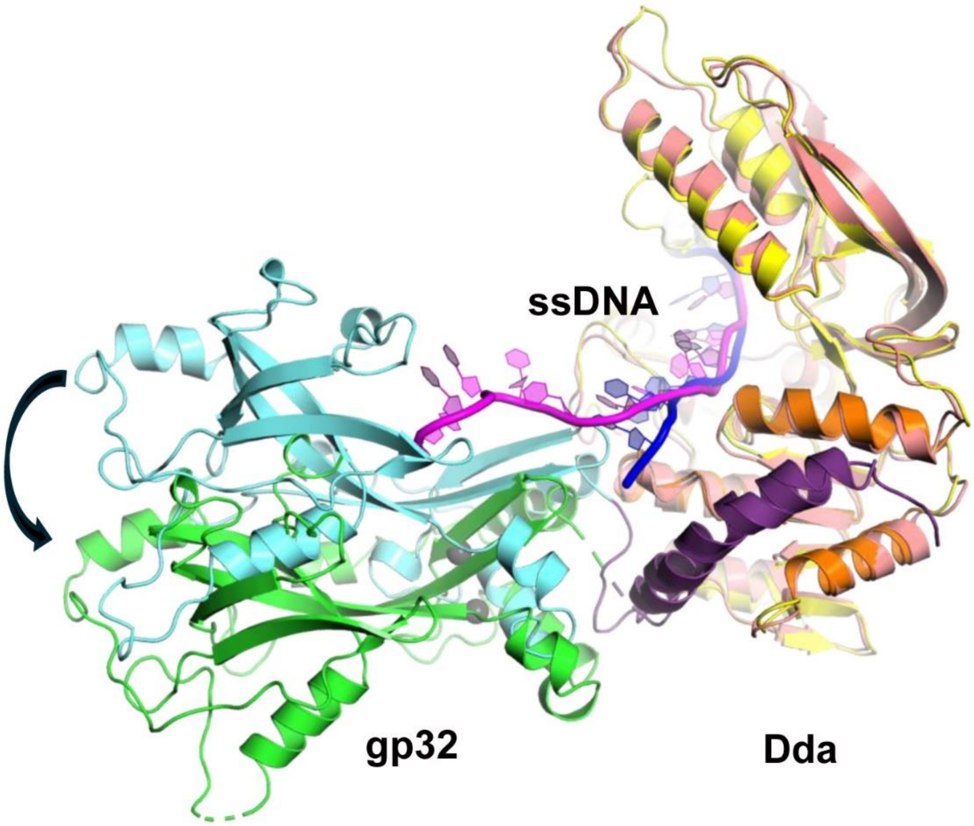

Figure S4. **Superposition of the two gp32-Dda-dT17 complexes in the crystal asymmetric unit.** Two complexes were overlayed on Dda to show that the relative orientations of the bound gp32 are slightly different as shown by the arrow. The components are colored the same as shown in Figure 1. Note that the orientation of the green gp32 appears to prevent the binding of its associated ssDNA emerging from Dda.

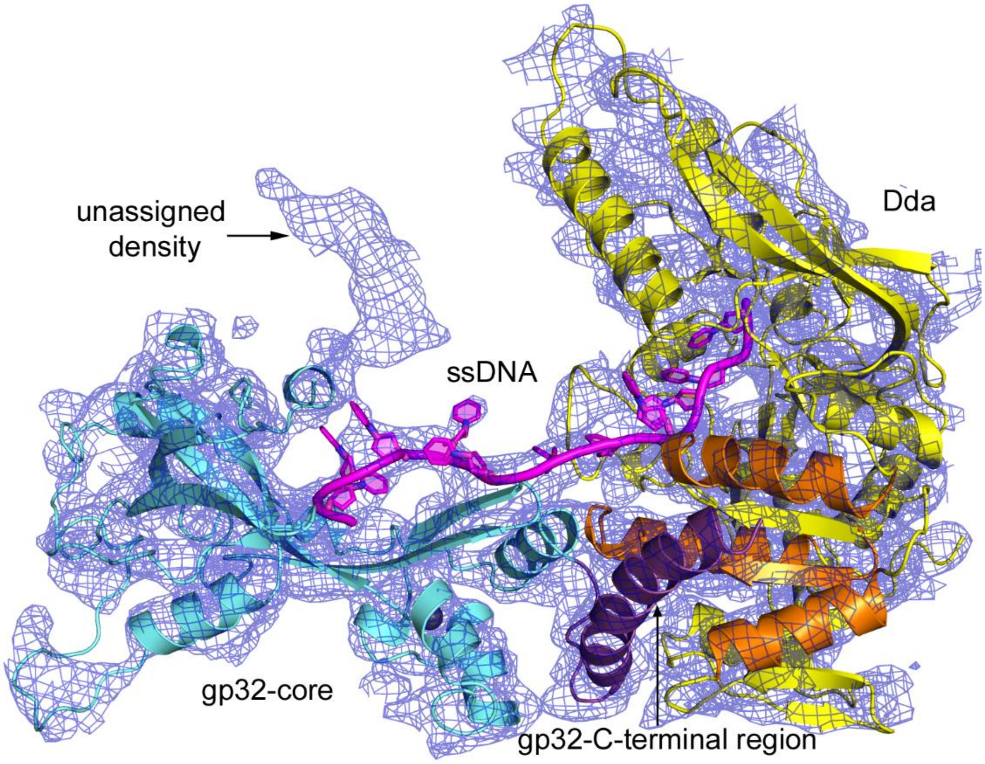

Figure S5. Electron density of the gp32-Dda-dT17 complex crystal structure. Only one of the complexes in the crystal asymmetric unit is shown and it corresponds to the complex shown in Figure 1B with the same orientation and coloring. The 2Fo-Fc map at 4.98 Å resolution is contoured at 1σ and shown in light blue mesh. The location of the unassigned density is consistent with the gp32 N-terminus that has not been modeled.

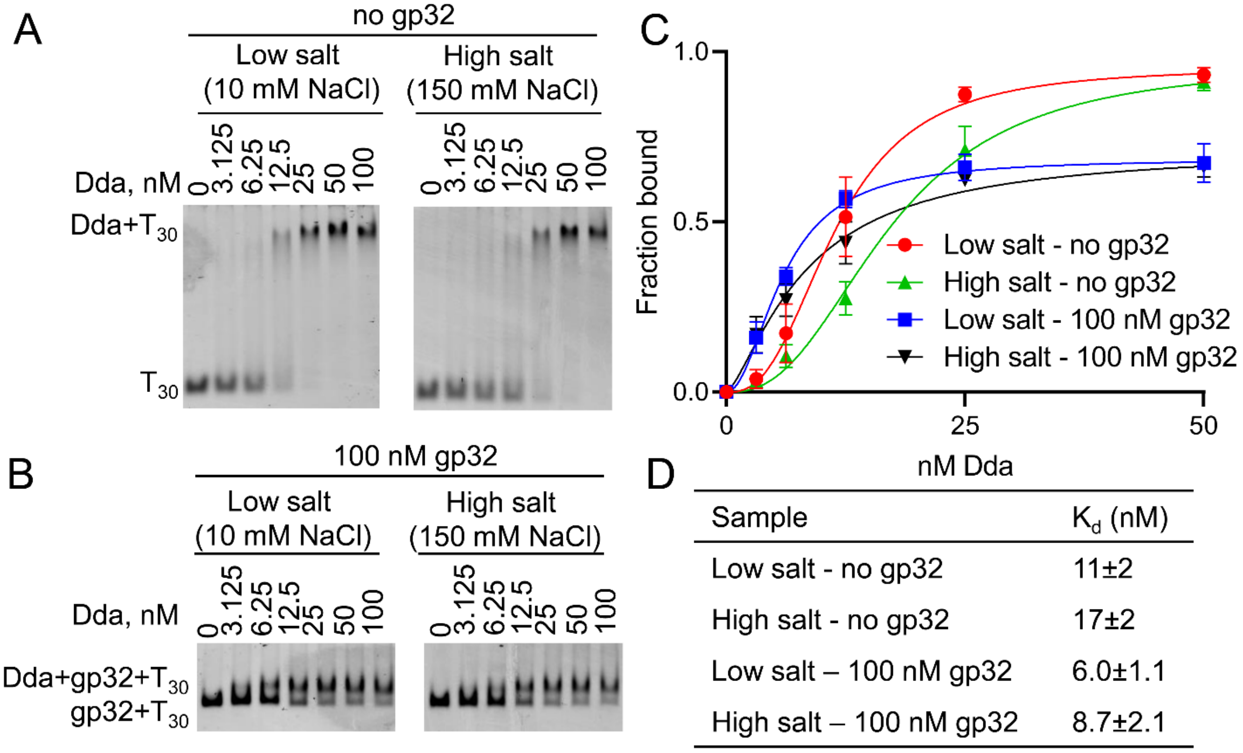

Sample	K_d (nM)
Low salt - no gp32	11±2
High salt - no gp32	17±2
Low salt – 100 nM gp32	6.0±1.1
High salt – 100 nM gp32	8.7±2.1

Figure S6. gp32 enhances the affinity of Dda for ssDNA. (**A**) Electrophoretic mobility shift assays (EMSAs) of 5 nM 5′-F-T_{30} with increasing concentrations of Dda at 10 mM NaCl and 150 mM NaCl. (**B**) EMSAs of 5 nM 5′-F-T_{30} with 100 nM gp32 and increasing concentrations of Dda at 10 mM NaCl and 150 mM NaCl. (**C**) The fraction of Dda bound DNA from three independent sets of EMSAs is plotted (average and standard deviation) and fit to the Hill equation. (**D**) The average and standard deviation of the apparent K_d for each condition are given.

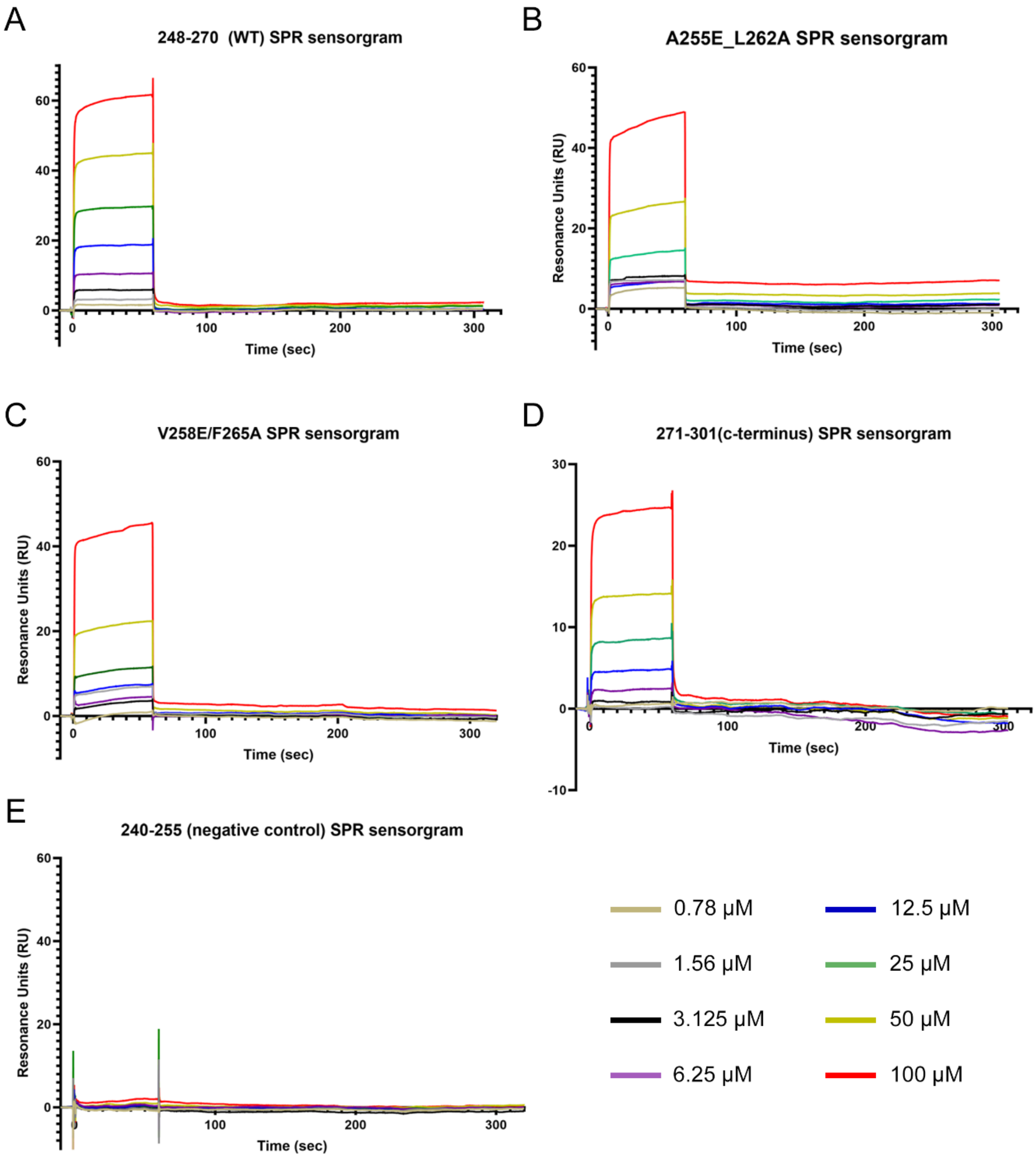

Figure S7. Sensorgrams from the SPR analysis 2 experiment. Responses at eight different concentrations. The curves represent the binding interactions over time, highlighting differences in association and dissociation rates for each concentration tested.

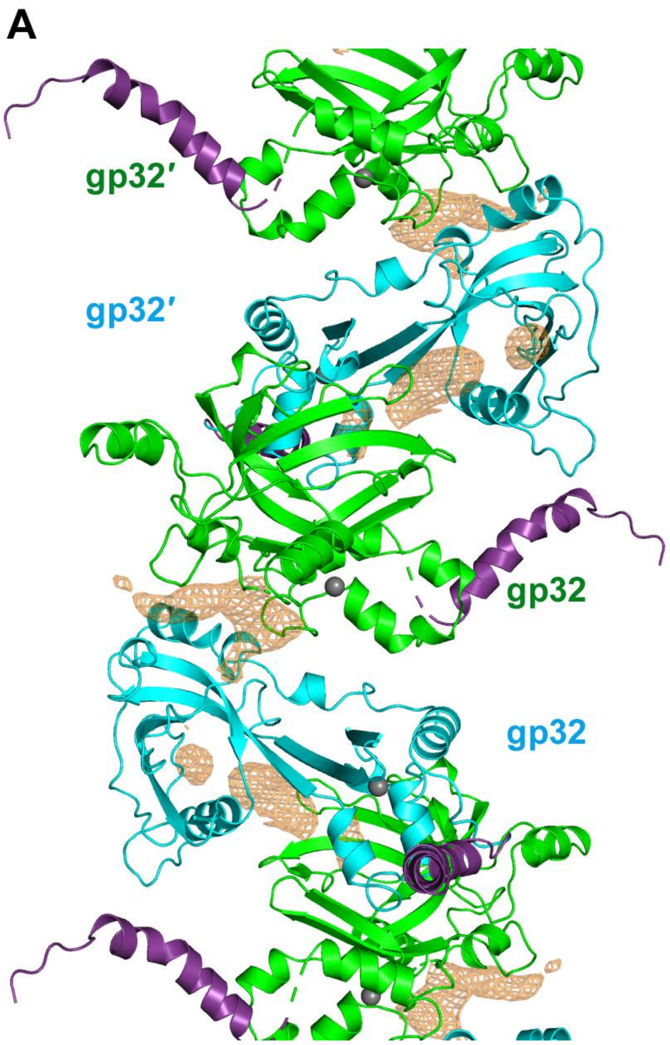

Figure S8. The gp32 filament in the gp32-Dda-dT17 complex crystal lattice. (A) Overall arrangement. The two gp32 proteins in the asymmetric unit (ASU) are colored cyan and green (see Figure 1A) and the Fo-Fc electron densities are consistent with their N-termini and shown as an orange mesh contoured at 2σ. The electron densities are consistent with the N-termini binding to the gp32 cores within and between asymmetric units. The C-terminal α-helices of gp32 that interact with Dda (not shown) are arrayed on the outside of the filament and colored purple. **(B)** The two independent gp32 dimers that create the filament; top – symmetry-related dimer; bottom – ASU dimer. In both dimers, the gp32 N-terminus interacts with its partner via the helix encompassing residues 141-152 (shown in grey), but the gp32 orientations are different. Symmetry-related gp32 proteins are indicated with a prime (‘).
